# From Black Stools to Gastrointestinal Stromal Tumor (GIST): A Case Report and Literature Review on This Unsuspected Danger

**DOI:** 10.7759/cureus.55172

**Published:** 2024-02-28

**Authors:** Emmanuel A Agyemang, Alexanderia S Williams, Nosike C Obionwu, Malaz Abdallah

**Affiliations:** 1 Internal Medicine, Newark Beth Israel Medical Center, Newark, USA

**Keywords:** mesenchymal tumors, imatinib therapy, gastrointestinal tumor (gist), gastrointestinal stromal tumor (gist), gastric gist

## Abstract

This case report presents the clinical details of a 42-year-old female without previous medical issues who presented with upper gastrointestinal bleeding (UGIB) characterized by melanotic stools. Initial examination revealed mild anemia and subsequent endoscopy identified a 4 cm submucosal gastric mass displaying recent bleeding indicators. Subsequent surgical pathology confirmed a high-grade gastrointestinal stromal tumor (GIST) of grade 2 with a heightened risk of recurrence. The significance of this case lies in underscoring the necessity of considering GIST in the differential diagnosis of UGIB, particularly among middle-aged individuals with no identifiable risk factors such as recent or chronic non-steroidal anti-inflammatory drug (NSAID) use, peptic ulcer disease, or alarm symptoms. Early detection and prompt surgical intervention assume paramount importance in enhancing patient outcomes. While complete resection stands as the cornerstone of treatment, adjuvant imatinib therapy is recommended for high-risk patients to mitigate the risk of recurrence.

## Introduction

Gastrointestinal stromal tumor (GIST) is recognized as a predominant mesenchymal tumor within the digestive tract, constituting less than 1% of all gastrointestinal malignancies [1]. Predominantly located in the stomach and intestines, it has an annual incidence of approximately 5000 cases in the United States [2]. The case under consideration pertains to a GIST presenting with an episode of upper gastrointestinal bleeding (UGIB).

## Case presentation

A 42-year-old female with no known significant past medical history presented to the emergency department for evaluation of dark tarry stools. She reported being in her usual state of health until two days before presentation when she noticed her stools had become black. However, she wasn't concerned until the morning of the presentation when her black stool was mixed with some blood, which prompted her visit. On direct questioning, the patient endorsed having occasional lightheadedness, dizziness, and easy fatigability. 

The physical examination was unremarkable, except for mild conjunctival pallor and tachycardia. Vitals were as follows: blood pressure of 103/72 mmHg, heart rate of 123 beats per minute, respiratory rate of 18 breaths per minute, and temperature of 98.7 °F. The impression of UGIB was made, hence a complete blood count (CBC) with repeats every two hours, a comprehensive metabolic panel (CMP), coagulation panel, *Helicobacter pylori *stool antigen test, and CT abdomen and pelvis with contrast were ordered. Initial CBC showed hemoglobin of 7.7 g/dl, a WBC count of 13.6 x 10^9, a platelet count of 266 x 10^9, CMP significant for mild hypokalemia (3.1), elevated blood urea nitrogen (BUN) of 21.0, and bilirubin of 0.1. The results of the repeat CBC are shown below in Table 1. 

**Table 1:** Serial laboratory test results

Parameters	Initial presentation	At two hours	At four hours
White blood cells	13.6 x 10^9	14.5	14.4
Hemoglobin	7.7 g/dL	6.8 g/dL	6.4 g/dL
Platelets	266 x 10^9	230	218
Blood urea nitrogen	21.0	N/A	N/A

**Figure 1:**
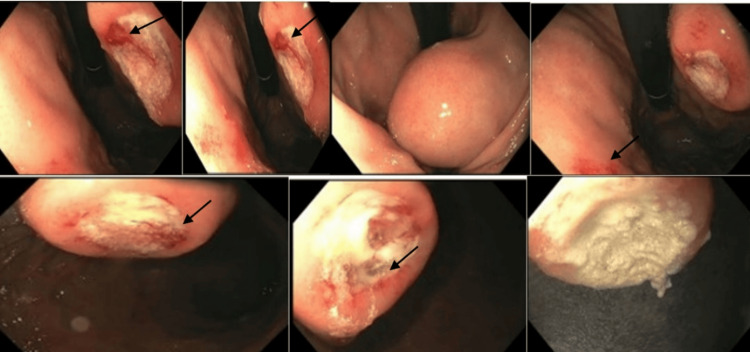
Upper GI endoscopy reveals a well-circumscribed submucosal mass in the gastric body with stigmata of recent bleeding

The patient was transfused with a unit of packed red blood cells and a stat consult was made to gastroenterology for endoscopy. Subsequent esophagogastroduodenoscopy and endoscopic ultrasonography, as well as CT, demonstrated a 4 cm well-circumscribed submucosal mass in the gastric body with stigmata of recent bleeding (Figure 1).

The patient's condition was subsequently discussed at the multidisciplinary tumor board meeting, and she was deemed to have resectable disease. Consent was obtained, and the patient underwent a laparoscopic partial gastrectomy, with the excised portions sent for pathological examination. Surgical pathology reported a tumor that measured 5.5 x 4 x 3 cm and had ulcerated the gastric mucosa. Immunostains showed the tumor cells were positive for CD117 and CD34 and negative for S100, smooth muscle antibodies (SMA), desmin, and beta-catenin, consistent with GIST, spindle cell type, and high-grade G2 (mitosis rate: 15; pT3 5 cm to 10 cm; stage 1b: high risk of recurrence). The patient was discharged home postoperatively on day 2, with plans to start oral imatinib 400 mg daily adjuvant therapy for the next three years to prevent recurrence and six monthly follow-up appointments scheduled at the oncology outpatient clinic.

## Discussion

Gastrointestinal stromal tumors are prevalent mesenchymal neoplasms within the digestive tract, comprising less than 1% of all gastrointestinal malignancies [1]. Coined in 1983, 'GIST' delineates an atypical non-epithelial tumor in the GI tract, devoid of conventional smooth muscle or Schwann cell characteristics [3]. Historically misclassified as leiomyomas, leiomyoblastoma, and leiomyosarcomas, molecular advancements have elucidated the origin of GIST from interstitial cells of Cajal (ICC), or precursor cells [4]. Distinctive features of GIST cells include the expression of CD117, leading to the identification of c-KIT, a membrane receptor protein with tyrosine kinase activity [3].

Globally, GIST incidence ranges from 10 to 15 cases per million, with approximately 5000 new cases diagnosed annually in the United States [5,6]. It predominantly affects older adults, peaking between 60 and 65 years old. Gastrointestinal stromal tumors exhibit an equitable distribution across sex, geography, race, and ethnicity, with cases before the age of 40 being rare [7,8]. While GISTs can emerge from any GI tract segment, the stomach (55.6%) prevails as the most common site, followed by the small intestine (31.8%), colorectal (6%), other locations (5.5%), and the esophagus (0.7%) [5,7].

Owing to their submucosal location and non-invasive nature relative to carcinomas, GISTs often remain asymptomatic until advanced stages [9]. Symptomatic presentations include GI bleeding, early satiety, abdominal distension, and discomfort or pain attributable to tumor compression [7,9]. Gastrointestinal bleeding manifestations encompass chronic insidious bleeding leading to anemia or acute, life-threatening episodes of melena or hematemesis [8].

The radiological and gross appearances of GISTs exhibit variability, encompassing intraluminal, intramural, and external components, as well as pedunculated extramural and cystic presentations [7]. Typically identified by endoscopy as submucosal tumors, pathological diagnosis primarily occurs post-surgery, revealing a mesenchymal neoplasm with spindle cell or epithelioid histology generally positive for KIT (CD117 leukocyte antigen) [7,9].

Prognosis in GIST is influenced by several factors, including mitotic index, tumor size, location (gastric vs. non-gastric), and tumor rupture [9]. The accepted criteria for malignancy prediction is mitotic activity exceeding 5 mitoses per 50 high-power fields (HPF) and a tumor size surpassing 5 cm, indicating heightened malignant potential [1]. Surgical resection stands as the fundamental treatment for localized GIST, with recurrence risks varying based on tumor size [10]. Adjuvant therapy with imatinib for three years is recommended for patients at significant risk of recurrence, improving both relapse-free survival and overall survival, particularly in high-risk cases and those with ruptured GISTs [1]. Ongoing research explores newer therapeutic modalities for GIST, including endoscopic ultrasound-guided alcohol injection and immunotherapies like nivolumab and ipilimumab [1].

## Conclusions

This report highlights the importance of considering GIST in the differential diagnosis of patients presenting with UGIB, especially middle-aged individuals with no significant past medical history. Early diagnosis and prompt surgical intervention are crucial for improving outcomes in GIST patients. While complete resection is the mainstay of treatment, adjuvant therapy with imatinib is recommended for high-risk patients to reduce the risk of recurrence.

## References

[1] Enodien B, Hendie D, Müller T, Taha-Mehlitz S, Frey DM, Taha A (2023). Gastrointestinal stromal tumor (GIST): a remarkable case report and literature review. Cureus.

[2] Sandler RS, Everhart JE, Donowitz M (2002). The burden of selected digestive diseases in the United States. Gastroenterology.

[3] Carvalho N, Albergaria D, Lebre R, Giria J, Fernandes V, Vidal H, Brito MJ (2014). Anal canal gastrointestinal stromal tumors: case report and literature review. World J Gastroenterol.

[4] Mantese G (2019). Gastrointestinal stromal tumor: epidemiology, diagnosis, and treatment. Curr Opin Gastroenterol.

[5] Kelly CM, Gutierrez Sainz L, Chi P (2021). The management of metastatic GIST: current standard and investigational therapeutics. J Hematol Oncol.

[6] Dupart J, Zhang W, Trent JC (2011). Gastrointestinal stromal tumor and its targeted therapeutics. Chin J Cancer.

[7] Miettinen M, Lasota J (2013). Gastrointestinal stromal tumors. Gastroenterol Clin North Am.

[8] Serrano C, Martín-Broto J, Asencio-Pascual JM (2023). 2023 GEIS Guidelines for gastrointestinal stromal tumors. Ther Adv Med Oncol.

[9] Nishida T, Blay JY, Hirota S, Kitagawa Y, Kang YK (2016). The standard diagnosis, treatment, and follow-up of gastrointestinal stromal tumors based on guidelines. Gastric Cancer.

[10] Jones RL (2014). Practical aspects of risk assessment in gastrointestinal stromal tumors. J Gastrointest Cancer.

